# CTHRC1 activates pro-tumorigenic signaling pathways in hepatocellular carcinoma

**DOI:** 10.18632/oncotarget.22164

**Published:** 2017-10-27

**Authors:** Yunpeng Wang, Mijin Lee, Goungran Yu, Hua Lee, Xueji Han, Daeghon Kim

**Affiliations:** ^1^ Division of Gastroenterology and Hepatology, Department of Internal Medicine, Chonbuk National University Medical School and Hospital, Jeonju, Jeonbuk, Republic of Korea; ^2^ Department of Infectious Disease, Yanbian University Hospital, Yanji, Jilin, China

**Keywords:** CTHRC1, HCC, CREB, Snail, metastasis

## Abstract

CTHRC1 expression is involved in invasion and metastasis in various tumors. However, the molecules involved in its signaling pathways in hepatocellular carcinoma (HCC) remain elusive. The migration and invasion abilities of HCC cells stably expressing CTHRC1 were assessed *in vitro* and *in vivo* with a mouse model. Moreover, signaling pathways involved in invasion and metastasis were analyzed. CTHRC1 was abundantly expressed in HCC cell lines and HCC tissues. CTHRC1 was also detectable in the serum of HCC patients, compared with non-tumor controls. CTHRC1 mRNA was positively correlated with large tumor size (*p* <0.003), Edmondson differentiation grade (*p* <0.0001), microvessel invasion (*p* <0.05), intrahepatic metastasis (*p* <0.005), and HCC stage (AJCC, *p* <0.0001). Ectopic expression of CTHRC1 in HepG2 cells promoted cell migration and invasiveness *in vitro*, and promoted tumor metastasis in a lung metastasis mouse model. Knockdown of CTHRC1 by short hairpin RNA (shRNA) in HCC cells suppressed migratory and invasive abilities. Growth factor-mediated CTHRC1 expression promoted cancer cell invasiveness and metastasis through activation of CREB/Snail signaling, which induced EMT change and MMPs expression. Therefore, CTHRC1 and its downstream molecules may be potential therapeutic targets for HCC invasion and metastasis.

## INTRODUCTION

Hepatocellular carcinoma (HCC) is a fatal liver cancer and a leading cause of cancer mortality worldwide. Its morbidity and mortality has risen in recent years [1, 2]. As invasion and metastasis are central causes of HCC-related death, identifying novel invasion and metastasis-related molecules is useful for predicting the prognosis and designing new treatments for advanced HCC.

Collagen triple helix repeat containing-1 (CTHRC1) contains a short collagen triple helix repeat NH2-terminal peptide and a COOH-terminal domain [3]. Expression of CTHRC1 promoted cell migration, furthermore, inhibited collagen I synthesis in rat fibroblasts, suggesting CTHRC1 may play a role in tissue repair in vascular remodeling by promoting cell migration and limiting collagen matrix deposition in response to injury [3]. Likewise, CTHRC1 transgenic mice revealed reduced neointimal lesion and adventitial collagen deposition after carotid artery ligation [4]. Recently, CTHRC1 have revealed to activate the planar cell polarity pathway through stabilizing the Wnt-receptor complex [5]. Previous studies showed overexpression of CTHRC1 in melanoma and cancers of many other organs [6]. Overexpression of CTHRC1 in hepatocellular carcinoma contributes to tumor invasion and predicts worse prognosis [7]. However, to date, few investigations have addressed the molecules involved in related signaling pathways, which promote HCC invasion and metastasis.

Matrix metalloproteinases (MMPs) play a role in cancer cell migration [8, 9]. The extracellular matrix (ECM) is critically involved in the modulation of MMPs in pathophysiological states [10, 11]. There is considerable evidence implicating MMPs in the degradation of ECM during the metastatic process. Secreted CTHRC1 overproduced in cancer cells may act on the surrounding microenvironment, such as stromal cells and ECM, to promote tumor invasion and metastasis.

In the present study, the functional role of CTHRC1 was investigated in HCC cell invasion and metastasis *in vitro* and in a mouse model, respectively. We found that growth factor-mediated CTHRC1 promoted invasion and metastasis in HCC cells through the PI3K/Akt/CREB(Snail)/MMP signaling pathways.

## RESULTS

### Expression of CTHRC1 in HCC tissues and cell lines

**Figure 1:**
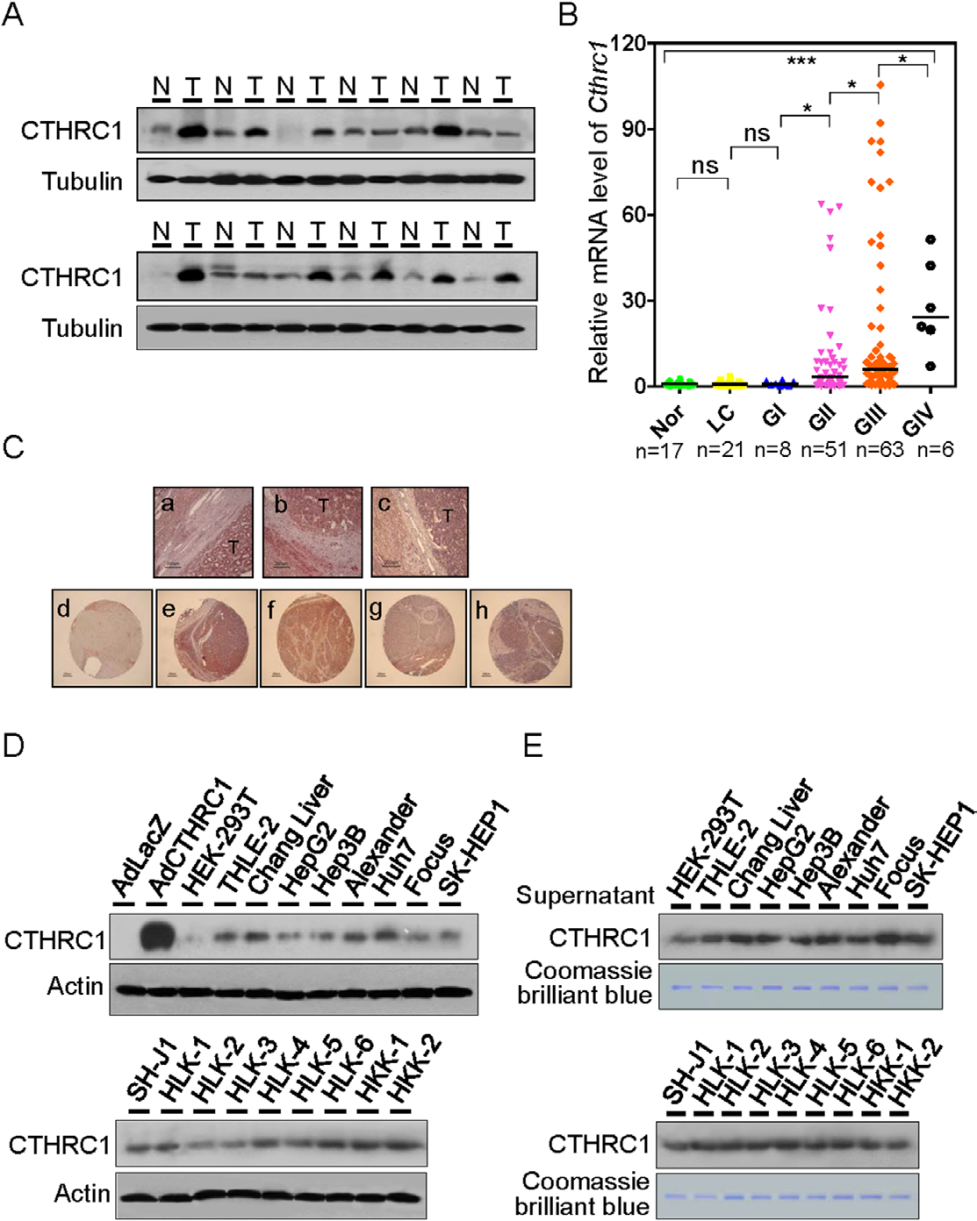
Expression levels of CTHRC1 in HCC tissue and cell lines **(A)** Western blot analysis of CTHRC1 expression in HCC tissues compared with paired corresponding non-tumorous tissues (T, tumor; N, non-tumor tissues). **(B)** CTHRC1 mRNA levels were measured in six groups: normal liver (NL, n = 17), liver cirrhosis (LC, n = 21), Edmondson grade I (GI, n = 8), Edmondson grade II (GII, n = 51), Edmondson grade III (GIII, n = 63), and Edmondson grade IV (GIV, n = 6). Bars indicate medians. Mann-Whitney U tests was used for the calculation of *P* values. The Kruskal-Wallis test was used for overall comparison. ^*^P < 0.05; ^***^P < 0.001. **(C)** Overview of CTHRC1-positive HCC tissues (a-c). CTHRC1 immunoreactivity was correlated in part with normal (d) and advancement of Edmondson differentiation (e-h). **(D)** Western blot analysis of CTHRC1 expression in HCC cell lines. Lysates of HepG2 cells transduced with adenovirus LacZ or CTHRC1 were used as negative or positive controls, respectively. **(E)** Western blot analysis of secreted CTHRC1 in HCC cell supernatant. Proteins detected by Coomassie Blue staining served as an internal control for protein loading.

CTHRC1 protein expression was determined by immunoblot analysis in 12 HCC/non-tumor tissue pairs (Figure 1A). CTHRC1 was highly expressed in HCCs (75%) compared with nontumor tissues. We further statistically analyzed CTHRC1 messenger RNA (mRNA) abundance with real-time RT-PCR in six relevant groups of samples: normal liver (NL), liver cirrhosis (LC), GI (Edmondson-Steiner grade I), GII, GIII, and GIV HCCs (Figure 1B). Levels of CTHRC1 mRNA significantly increased according to HCC differentiation status. We also asked if there were correlations between CTHRC1 expression and clinicopathological variables in HCC (Supplementary Table 1). Intriguingly, large tumor size, Edmondson-Steiner differentiation grade, microvessel invasion, intrahepatic metastasis, and stage (AJCC) were the variables that showed significant differences between subgroups.

Paraffin sections of CTHRC1 tissues were stained with the immunoperoxidase method for the expression of CTHRC1 as described in the Supplementary Materials and Methods. Representative fields were photographed at 200 × magnification (Figure 1C, a-c). IHC findings were observed according to tumor differentiation in tissue microarrays (TMA) from an independent cohort of patients (Figure 1C, d-h); CTHRC1 expression was barely detected in normal tissue (Figure 1C, d), but moderate and strong expression was seen in HCC with poor differentiation (Figure 1C, e-h).

Expression of CTHRC1 was also determined in a panel of HCC cell lines (Figure 1D). CTHRC1 was highly expressed in most cell lines, and weakly expressed in HepG2, HLK-2, and HLK-3 cells. The protein levels of secreted CTHRC1 in cell supernatants were measured with Western blotting (Figure 1E). CTHRC1 levels were detected in the serum of HCC patients compared with normal control serum (Supplementary Figure 1A). The levels of CTHRC1 in the serum of HCC patients decreased after surgery (Supplementary Figure 1B). Intracellular CTHRC1 existed as a 26 kDa protein, corresponding to the monomeric CTHRC1, as determined by reducing and non-reducing SDS–PAGE (Supplementary Figure 2). In contrast, secreted CTHRC1 existed in multiple forms as shown by non-reducing gel, consisting of 26, 50, and 75, kDa species. These bands correspond to the monomer, homodimer, and homotrimer sizes respectively. Our data suggest that HepG2 cells infected with CTHRC1-expressed adenovirus secreted CTHRC1 protein into the medium where it formed homo- and heterodimeric complexes (Supplementary Figure 3). In order to determine the specific immunoreactivities of CTHRC1, HEK-293T cells transfected with GFP-tagged or Myc-tagged CTHRC1 were detected using Western blot analysis. Anti-CTHRC1 antibody specifically recognized the band corresponding to the CTHRC1 protein (Supplementary Figure 4). Next, HLK-3 and HepG2 cells were transfected with the GFP-tagged CTHRC1 or empty vector. IF assay revealed that the ectopic expression of GFP-tagged CTHRC1 was mainly localized in the cytoplasm, but endogenous CTHRC1 was seen in both the nucleus and cytoplasm (Supplementary Figure 5).

### Effect of CTHRC1 on cell proliferation

**Figure 2:**
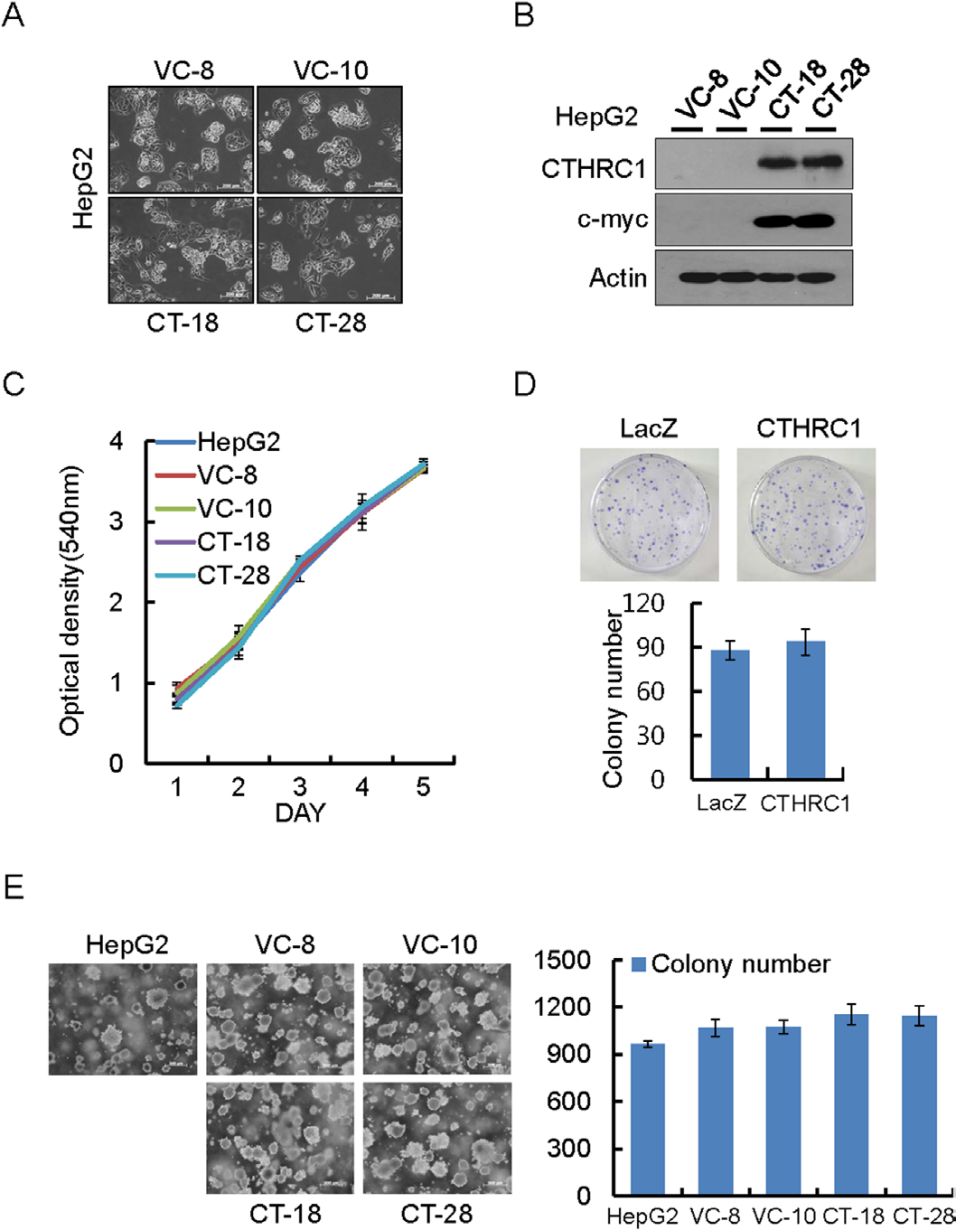
Proliferation ability of HepG2 cells by ectopic expression of CTHRC1 **(A)** Morphological changes in CTHRC1-expressed HepG2 cells and vector control cells. **(B)** CTHRC1 expression was confirmed by immunoblot analysis in HepG2 cells stably expressing CTHRC1 and vector control cells. **(C)** Proliferation ability by CTHRC1 in the stable transfectants. Cell proliferation was measured by MTT assay 5 days after the cells were seeded on 24-well plates (2 × 10^4^ cells/well). Each value is mean ± SEM from 3 independent experiments. **(D)** Representative dishes by colony-forming assay after CTHRC1 transfection and selection for 2-3 weeks in HepG2 cells. Each value is mean ± SEM (n = 3). **(E)** CTHRC1 transfectants (2 × 10^5^) were plated per 60-mm dish and cultured in 0.3% soft agar in DMEM plus 10% FBS for 21 days. Each value is mean ± SEM (n = 3).

HepG2 cells which barely express CTHRC1 were used to establish stable cells expressing CTHRC1. Morphological changes in HepG2 cells were observed under phase-contrast microscope. Cells expressing CTHRC1 were less adhesive and more migratory than parent cells (Figure 2A and 2B). MTT assay showed that overexpression of CTHRC1 had no effect on cell proliferation (Figure 2C). Similarly, the colony generation ability of CTHRC1 overexpressed cells was also not different from their parent cells as assessed by colony formation assay (Figure 2D) and soft agar assay (Figure 2E).

### CTHRC1-mediated invasion and metastasis ability of HCC cells

**Figure 3:**
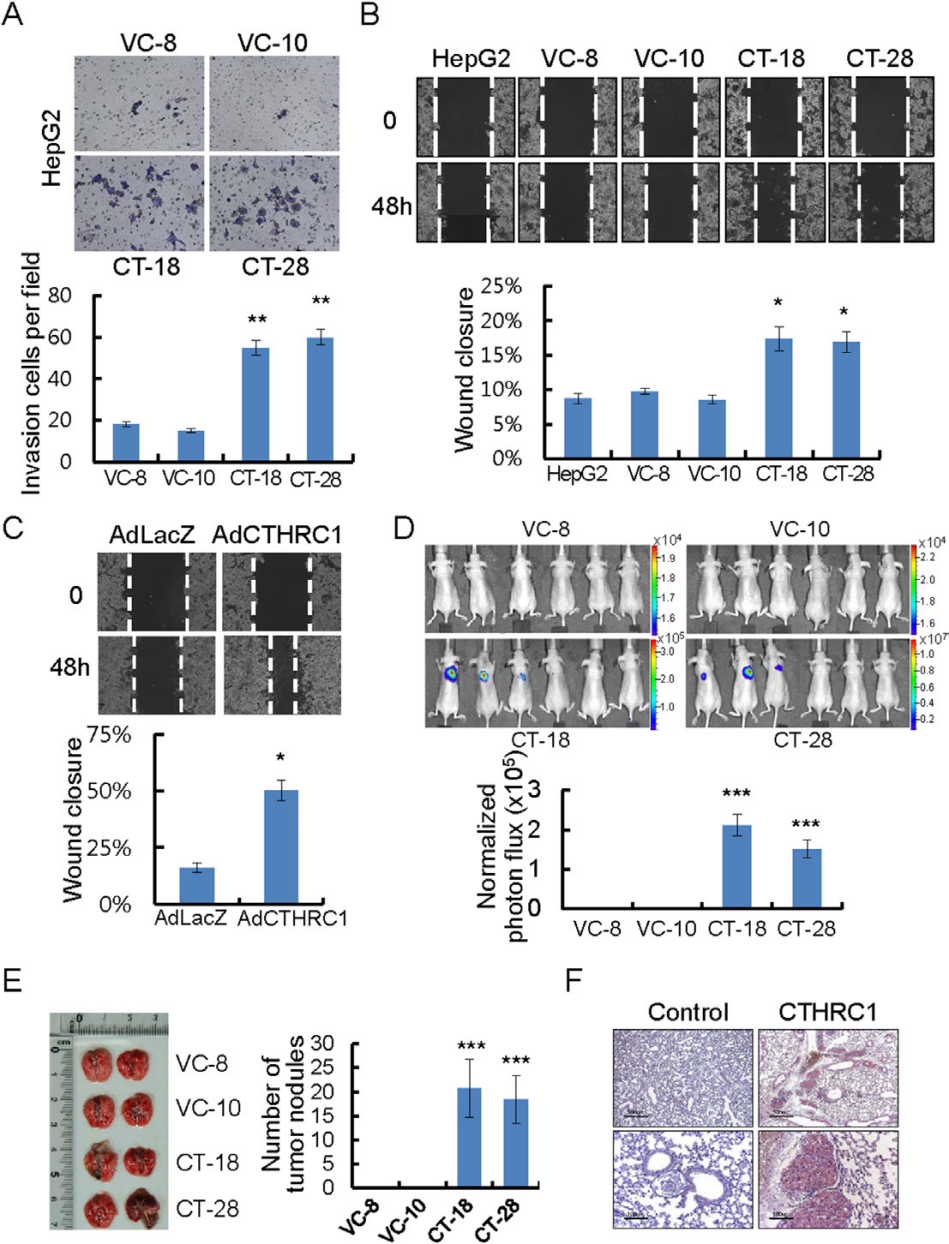
CTHRC1-mediated promotion of invasion and migration in HCC cells **(A)** Photomicrographs of a Matrigel Boyden chamber showing more colonies of CTHRC1-expressed HepG2 cells than vector control cells. HCC cells invaded were normalized to the cell mass on the bottom sides of the membrane (n = 3, mean ± SEM). ^**^P < 0.01. **(B)** HepG2 cells stably expressing CTHRC1 were then allowed to migrate into the wound area. Quantitative measurements of wound closure ability are shown (n = 3, mean ± SEM). ^*^P < 0.05. **(C)** The wound closure capacity of HepG2 cells after infection with AdLacZ or AdCTHRC1. Quantitative measurements of wound closure ability are shown (n = 3, mean ± SEM). ^*^P < 0.05. **(D)** HepG2 stable expressing CTHRC1 cells after infection Adeno-luciferase at 100 MOI 24hr, then 5×10^5^ cells via the tail vein to node mice. Five weeks after tumor implantation, bioluminescent images demonstrated that CTHRC1-expressing tumor cells spread to the both lungs (n = 6), representative anterior-posterior images, 35 s exposure time. Quantities are the measured means of photon flux (lower panel). ^***^P < 0.001. Color bar (Photons/Sec/cm^2^, Max/Min); VC-8, 1.96×10^4^/1.51×10^4^; VC-10, 2.31×10^4^/ 1.51×10^4^; CT-18, 3.46×10^5^/1.88×10^4^; CT-28, 1.19×10^7^/6.91×10^5^. **(E)** Fifty-five days after tumor implantation, mice were euthanized and their lungs evaluated for tumor nodules. Each value is mean ± SEM, ^***^P < 0.001. **(F)** Immunohistochemical staining of CTHRC1 in representative tumor tissue samples. CTHRC1 expression in representative tumor tissue samples from mice implanted with cells stably expressing CTHRC1 and with control cells. Scale bars, 500 μm (upper panel) or 100 μm (lower panel).

In a modified Boyden chamber assay, stable transfectants penetrated the matrix and colonized the bottom surface of the matrigel-coated membrane to a greater extent than vector control cells (Figure 3A). They also showed rapid wound closure ability, compared with vector control cells (Figure 3B). Similarly, the wound closure ability of adeno-associated virus expressing CTHRC1 was significantly higher than adeno-associated virus expressing LacZ (Figure 3C). The wound edge in each CTHRC1-expressing cell was more migratory compared with vector controls. HepG2 stably-expressing CTHRC1 cells infected with 100 multiplicity of infection (MOI) adeno-luciferase virus were examined after injection into the tail veins (200 μl of 5×10^5^ cells/mL) of nude mice. Sufficient bioluminescence data were collected at 5 weeks post-injection (Figure 3D). HepG2 stably-expressing CTHRC1 cells formed many metastatic nodules in both lungs of mice, but control cells formed few metastatic nodules (Figure 3E). An immunohistochemical examination revealed high expression of CTHRC1 in the metastatic nodules (Figure 3F). These results suggested that CTHRC1 expression played a important role in tumor invasion and metastasis in HCC cells.

### Downstream CTHRC1 signaling

**Figure 4:**
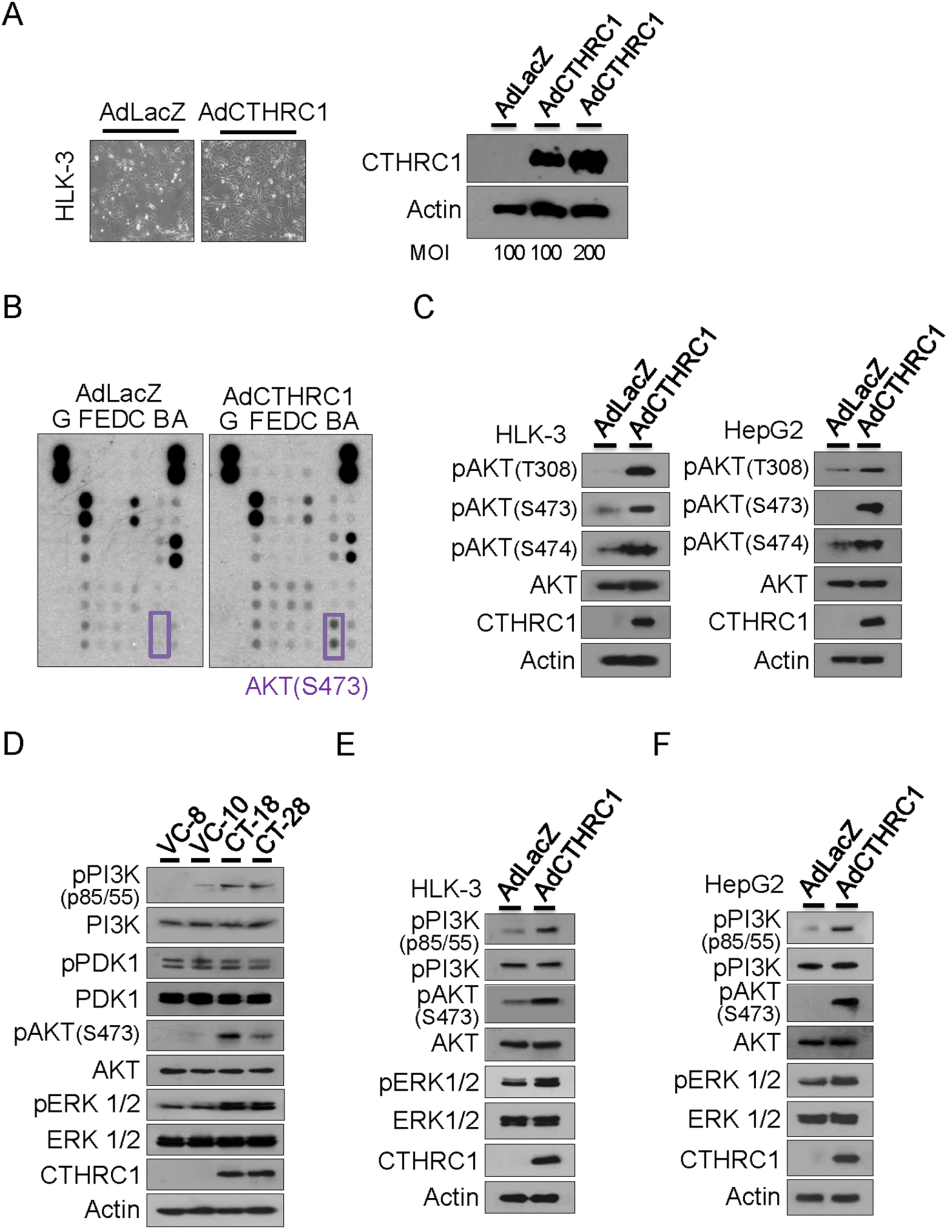
Downstream signaling of CTHRC1 **(A)** Adeno-associated virus expressing CTHRC1 or LacZ was transiently transduced into HLK-3 cells at a multiplicity of infection (MOI) of 100-200. Forty-eight hours after infection, overexpression of CTHRC1 in HLK-3 cells was confirmed by western blot analysis. **(B)** The signaling protein molecules are indicated in the box. The two doublets in the corners are used as internal positive controls. **(C)** Phosphorylation of Akt was measured by immunoblot after AdCTHRC1 or AdLacZ transduction into HLK-3 and HepG2 cells. The results shown are from three independent experiments. **(D)** Activation of PI3K, PDK, Akt, and ERK in the stable CTHRC1 transfectants compared to vector control cells. **(E)** Activation of PI3K, Akt, and ERK in the adeno-associated virus expressing CTHRC1 compared to mock control cells in HLK-3 cells or (F) in HepG2 cells.

Adeno-associated virus expressing LacZ (control) or CTHRC1 was transiently transduced into HLK-3 cells. Forty-eight hours after infection, overexpression of CTHRC1 in HLK-3 cells was confirmed (Figure 4A). Next, we searched for phosphorylation of downstream molecules using human phospho-kinase arrays (Figure 4B). CTHRC1 overexpression led to phosphorylation of Akt, as confirmed by immunoblot analysis. Akt phosphorylation occurred at T308, S473, and S474 in both HLK-3 and HepG2 cells (Figure 4C). Therefore, we examined Akt upstream and downstream molecules and found that CTHRC1 caused phosphorylation of PI3K as well as downstream ERK, but not PDK1 in the HepG2 tranfectants (Figure 4D). Adenovirus CTHRC1 expression also led to phosphorylations of PI3K, Akt and ERK in HLK-3 (Figure 4E) and HepG2 cells (Figure 4F). Therefore, CTHRC1 initially activated PI3K and subsequently activated its downstream signaling pathway.

### CTHRC1 knockdown inhibited migration and invasion ability in SH-J1 cells

**Figure 5:**
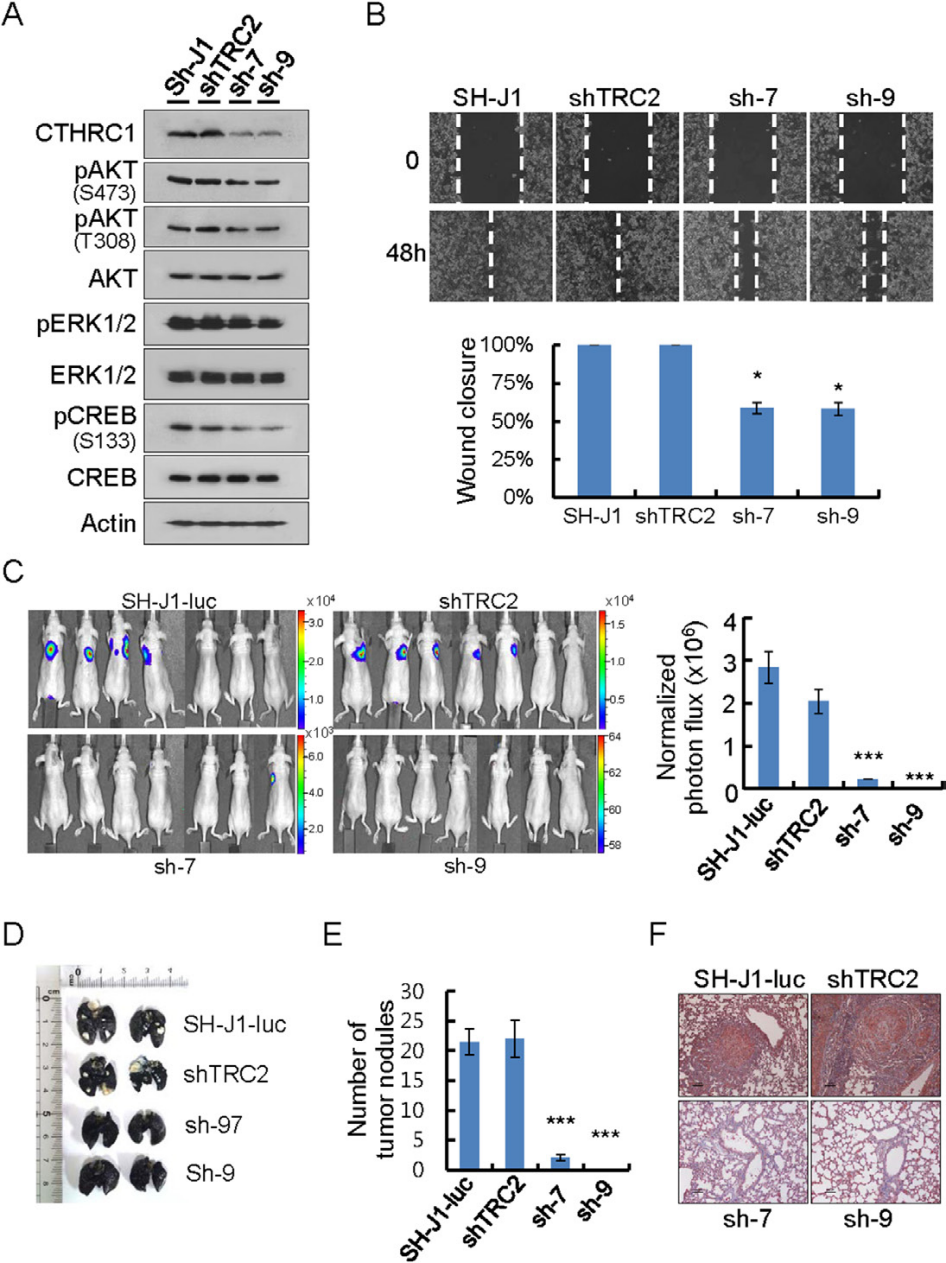
CTHRC1-mediated promotion of invasion and metastasis *in vitro* and *in vivo* **(A)** Protein lysates were prepared from cells transduced with lentiviral shnon-target (shTRC2) or shCTHRC1 (sh1997 and sh1999). Membranes were incubated with the indicated antibodies. **(B)** Wound closure capacity of SH-J1 cells with CTHRC1 knockdown was measured by wound healing assay. Quantitative measurements of wound closure ability are shown (n = 3, mean ± SEM). ^*^P < 0.05. **(C)** SH-J1-luc cells transduced with lentiviral shnon-target (shTRC2) or shCTHRC1 (sh1997 and sh1999), then 5 × 105 cells via the tail vein to nude mice. Five weeks after tumor implantation, Bioluminescent images demonstrated that CTHRC1- expressing tumor cells spread to the lungs (n = 7 mice/group, representative anterior-posterior images, 30 s exposure time). Quantities are the measured means of photon flux (right panel). Mean ± SEM. ^***^P < 0.001. Color bar (Counts, Max/Min); SH-J1-luc, 33106/2315; shTRC2, 16669/1107; sh-7, 7194/614; sh-9, 64/58. **(D)** Tumor-bearing lungs were inflated with India ink to detect tumor nodules. **(E)** Fifty-five days after tumor implantation, mice were euthanized and their lungs were evaluated for tumor nodules. Each value is mean ± SEM, ^***^P < 0.001. **(F)** Immunohistochemical staining of CTHRC1 in metastatic nodules. Scale bars, 100 μm.

The ERK/CREB pathway was reported to be involved in FoxM1-mediated hepatoma cell invasion and lung metastasis [12]. CREB also functions in the signaling pathway between Akt and MMPs in lung cancer cells [13]. Therefore, we established CTHRC1 knockdown cells by lentiviral delivery of CTHRC1 shRNA into metastasis-prone HCC cells (SH-J1) which express high levels of CTHRC1 and examined the Akt/ERK/CREB pathway.CTHRC1 knockdown inhibited activations of CREB, Akt, and ERK1/2, in comparison to parent and non-target cells (Figure 5A). The wound closure capacity of SH-J1 cells after CTHRC1 knockdown was slower than non-target cells (Figure 5B). The metastatic phenotype of the SH-J1-luc cells was assessed by injection into the tail veins (200 μl of 5 × 105 cells/mL) of nude mice. Sufficient bioluminescence data were collected at 8 weeks post-injection (Figure 5C). The parent and non-target cells were initially colonized and then continued growing into the both lungs with many metastatic nodules (Figure 5D and 5E). A histological examination demonstrated that injection of the parent and non-target cells resulted in numerous CTHRC1-positive metastatic lesions (Figure 5F).

### Acquisition of EMT characteristics by CTHRC1

**Figure 6:**
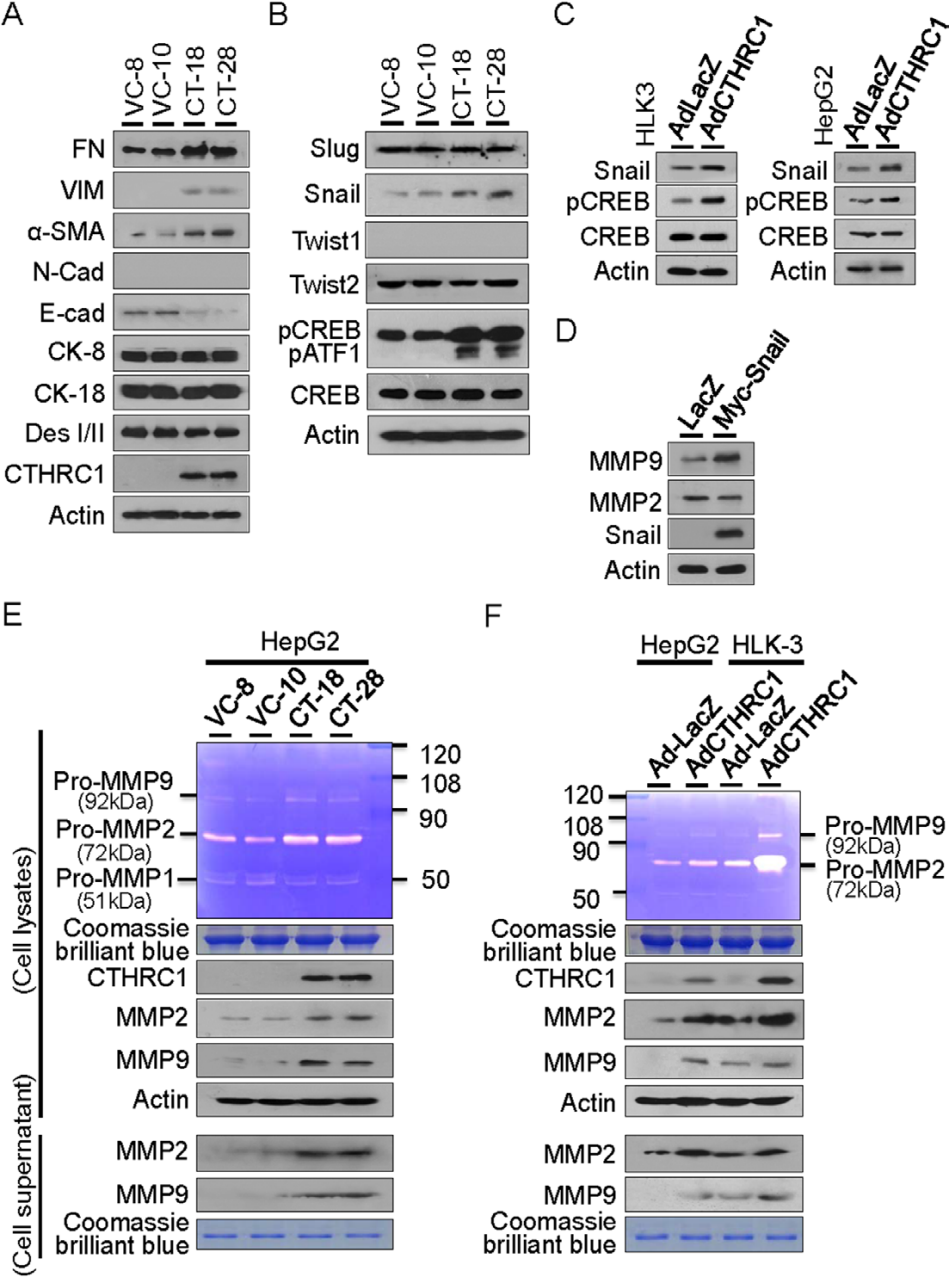
CTHRC1-mediated EMT characteristics and MMP expression **(A)** Western blot analyses showed acquisition of mesenchymal markers and loss of epithelial marker proteins in the CTHRC1 transfectants compared with vector control cells. FN, fibronectin; VIM, vimentin; α-SMA, alpha smooth muscle actin; N-cad, N-cadherin; E-cad, E-cadherin; CK, cytokeratin; Des, desmoplakin. **(B)** Snail expression and CREB activation were demonstrated in HepG2 cells stably expressing CTHRC1, other transcription factors Slug, Twist1 and Twist2 were not changed. **(C)** Snail expression and CREB activation were also observed in HLK-3 and HepG2 cells transduced with CTHRC1 expressing adenovirus for 48 hours. **(D)** MMP9, but not MMP2, was up-regulated by transient transfection with Snail in HepG2 cells. **(E)** Conditioned medium was collected and concentrated by centrifugation. Zymogram gel assays was performed. The gelatinolytic activity of MMP2 and MMP9 was assessed by Coomassie blue staining. The protein levels of MMP2 and MMP9 in stable CTHRC1 transfectants, compared with vector control cells checked by Western blotting. **(F)** MMP2 and MMP9 activity and expression from the HLK-3 and HepG2 cells transduced with AdCTHRC1 or AdLacZ were measured by zymogram gel assays and Western blotting.

The EMT of epithelial cancers is associated with aggressive tumors that are critical in HCC [14–17]. We therefore examined whether CTHRC1 overexpression was involved in tumor invasiveness and metastasis by changing the EMT phenomena in HCC cells. We found that CTHRC1 effectively promoted the expression of mesenchymal markers such as fibronectin (FN), vimentin (VIM), N-cadherin (N-cad), and alpha-smooth muscle actin (α-SMA). In contrast, CTHRC1 overexpression decreased the expression of epithelial markers, E-cad and desmoplakin I/II (DesI/II), but did not change cytokeratin 8 (CK-8) and 18 (CK-18) expression (Figure 6A). Either CREB or Snail knockdown inhibited the expression of mesenchymal markers, but increased the expression of epithelial markers compared to control cells (Supplementary Figure 6).

Snail transcriptional factor was reported to promote migration and invasion in HCC [18–20]. Accordingly, we found that Snail protein expression was significantly up-regulated in CTHRC1 transfectants. In contrast, the expression of Twist1, Twist2, and Slug did not change. Our results showed that activations of CREB and closely related protein ATF1 expression (activating transcription factor 1, can be detected with the same antibody) were prominently detected in stable CTHRC1-transfectants (Figure 6B), which suggests that CREB activation is downstream of CTHRC1. Snail expression was increased in HLK-3 and HepG2 cells transduced with Ad-CTHRC1 (Figure 6C). MMP9, but not MMP2, was up-regulated by transient transfection with Snail in HepG2 cells (Figure 6D) compared with vector controls. In contrast, CTHRC1 expression up-regulated both MMP2 and MMP9 in HepG2 (Figure 6E) and HLK3 cells (Figure 6F). Transcriptional promotion assay showed CTHRC1 efficiently enhanced both MMP2 and MMP9 promoter activities in HepG2 cells (Supplementary Figure 7). These results suggested that CTHRC1 over-expression played a crucial role in HCC metastasis through the Snail and CREB signaling pathways that modulate MMP expression.

### CREB, upstream molecule of MMP activation

**Figure 7:**
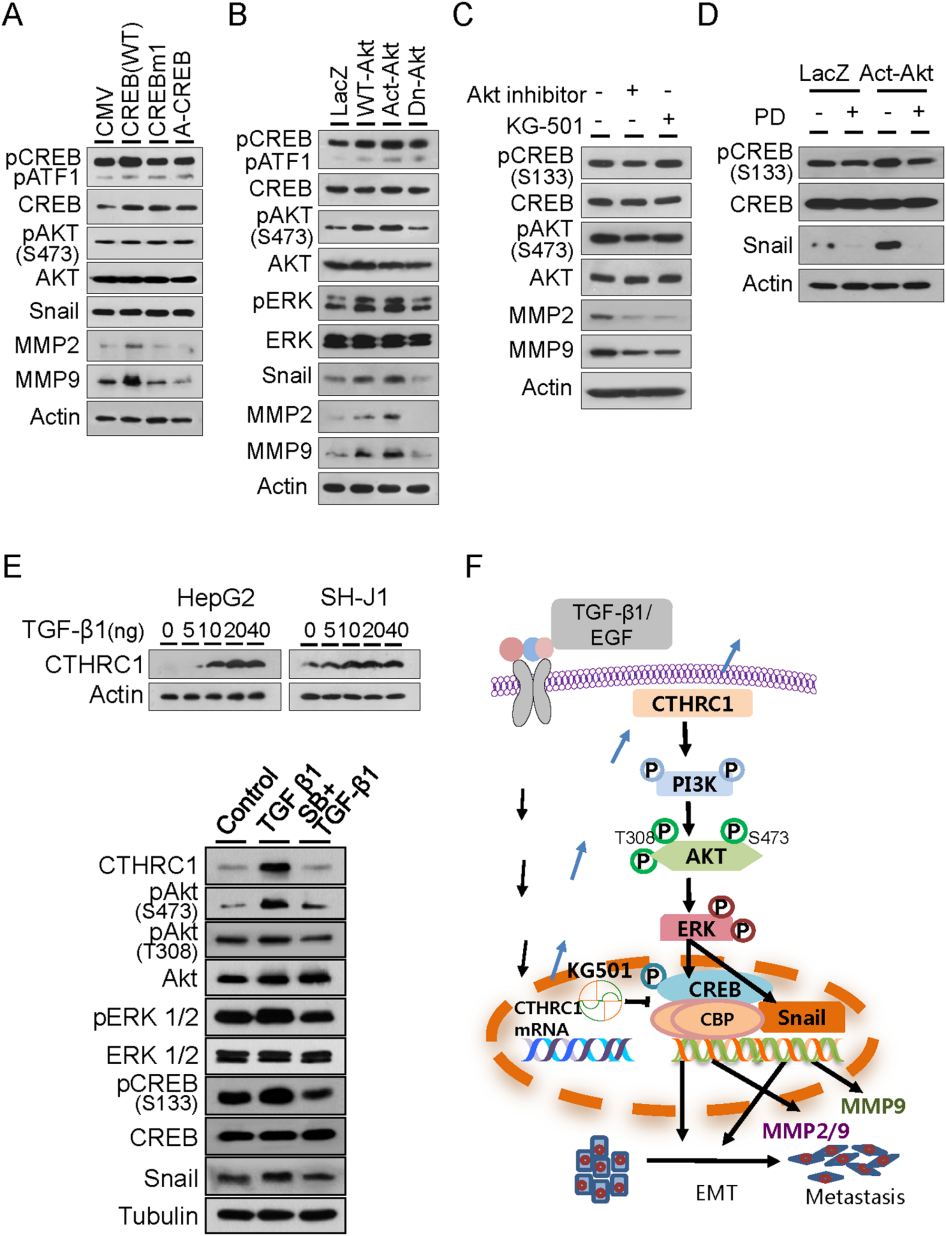
Downstream signaling of CTHRC1 **(A)** Immunoblot analysis of phospho-Akt, phospho-CREB, snail, MMP2 and MMP9 levels in HepG2 cells transiently transfected with control plasmid CMV, WT-CREB, CREBm1, or A-CREB. **(B)** Immunoblot analysis of phospho-Akt, phospho-ERK, phospho-CREB, Snail, MMP2 and MMP9 levels in HepG2 cells transiently transfected with LacZ, WT-Akt, Act-Akt, or Dn-Akt. **(C)** Phospho-Akt, phospho-CREB, Snail, MMP2 and MMP9 expression were measured after treatment with an Akt inhibitor or CREB inhibitor KG501 in SH-J1 cells. **(D)** CREB activation and Snail expression were measured after treatment with vehicle or ERK inhibitor PD98059 (PD) in HepG2 cells. **(E)** TGF-β1-induced CTHRC1 expression in HepG2 and SH-J1 cells. Cells were treated with TGF-β1 at the indicated concentrations for 4 days (upper panels). SB431542 suppressed TGF-β1-mediated activation of the CTHRC1/CREB (Snail) signaling pathway. HepG2 cells were treated with TGF-β1 (20 ng/ml) alone or in combination with SB431542 (SB, 10 μM) for 3 days (lower panels). **(F)** Schematic model of growth factor-mediated CTHRC1 expression. Invasiveness and metastasis of cancer cells are promoted through activation of the PI3K/Akt/ERK/CREB (Snail) signaling pathway, which induced EMT change and MMP2/MMP9 expression.

In order to investigate how CTHRC1 regulated MMP expression, HepG2 cells were transfected with a wild-type CREB (WT) and a mutant form of CREB (CREBm1) in which the transcriptional regulatory residue, serine 133, was mutated to alanine, and A-CREB, a potent inhibitor of CREB DNA binding activity. MMP2 and MMP9 expression were up-regulated by WT-CREB but not by CREBm1 or A-CREB. Akt activation and Snail expression were not changed by WT-CREB (Figure 7A). This suggested that CREB was downstream of Akt and upstream of MMP.

HepG2 cells were transfected with expression plasmids of wild-type (WT)-Akt, activated (Act)-Akt, or its dominant negative form (Dn-Akt). The results showed that ERK1/2 and CREB were efficiently phosphorylated by WT-Akt and Act-Akt but not Dn-Akt. Expressions of Snail, MMP2, and MMP9 were also increased by WT-Akt and Act-Akt (Figure 7B). MMP2 and MMP9 expression were reduced by Akt inhibitor and CREB inhibitor KG-501 in SH-J1 cells, as previously reported [21]. Akt phosphorylation was not changed after treatment with KG501, suggesting that Akt was downstream of CTHRC1 and up stream of CREB and modulated MMP2 and MMP9 expression (Figure 7C). Addition of the ERK inhibitor PD98059 in the culture medium suppressed CREB activation and Snail expression in the presence or absence of Act-Akt (Figure 7D). This suggested that ERK was upstream of CREB activation and Snail expression that modulated MMP2 and MMP9 expression. We also found that TGF-β1 or EGF upregulated CTHRC1 expression in a dose-dependent manner in HepG2 and SH-J1 cells (Figure 7E and Supplementary Figure 8). TGF-β1 inhibitor SB431542 efficiently suppressed CTHRC1 expression and inhibited activations of their downstream molecules, implicating that growth factors may be responsible for CTHRC1 expression and subsequent tumor invasion and metastasis (Figure 7E and 7F).

## DISCUSSION

CTHRC1 has shown to be abundantly expressed in human pancreatic cancer tissues [22], hepatocellular carcinoma [7], gastric cancer [23], colorectal cancer [24], and gastrointestinal stromal tumors [25]. Accordingly, we found that CTHRC1 protein levels were higher in HCC tissues than in corresponding nontumor tissues. Secreted CTHRC1 was detected in HCC patient serum. As expression of CTHRC1 was correlated with poor prognosis [7], our data also showed that CTHRC1 was highly expressed according to larger tumor size and worse differentiation. These results suggested that CTHRC1 played a critical role in HCC tumor progression.

Besides *in vitro* migration and invasion assays, an orthotopic xenograft model of HepG2 cells transduced with Ad-CTHRC1 indicated that its expression led to more aggressive behavior and metastases. It has been reported that CHTRC1 acts as a Wnt cofactor which selectively activates the planar cell polarity (PCP) pathway [5]. CTHRC1 promoted tumor cell migration and invasion by activating Wnt/PCP signaling supported by activating Rac1 in pancreatic cancer [22], activating RhoA in HCC [7] and activating both Rac1 and RhoA in GIST cells [25]. Moreover, CTHRC1 promoted colorectal cancer cell invasiveness through activation of ERK and subsequent induction of MMP9 expression [26]. We found that overexpression CTHRC1 in HepG2 cells led to ERK-CREB/Snail activation and expression, while knockdown of CTHRC1 resulted in inhibition of either signaling pathway in SH-J1 cells.

It has been shown in several cancer types that various factors induced high expression of MMP2 and MMP9; ionizing radiation promoted hepatocellular carcinoma cell invasion with an increase in MMP9 expression [27], enhancement of Bcl-2 expression increased gastric cancer cell invasion with an increase in MMP2 expression and activity [28], insulin-like growth factor-1 (IGF-1) induced invasion of distinct breast cancer cells (SUM-159-PT and MDA-MB-231) with induction of MMP proteins [29], and hepatitis B viral HBx protein increased hepatocellular carcinoma invasion potential with induction of MMP9 gene expression [30]. Our results also defined the role of CHTRC1 as an inducer of EMT characteristics in HepG2 cells through activation of the CREB and Snail pathways. CTHRC1 also increased the activities of MMP2 and MMP9. Both MMP proteins can be detected in lysate and cell supernatant of stable or transient HepG2 and HLK-3 cell CTHRC1-transfectants. A promoter assay revealed that CTHRC1 effectively increased both MMP2 and MMP9 promoter activities. These accumulating data indicated that CTHRC1 was a key regulator of HCC invasion and metastasis through EMT change and modulation of MMP expression in the tumor microenvironment.

To further determine the CTHRC1 signaling pathway relationship to HCC invasion and metastasis, we explored the Akt signaling cascade and its upstream and downstream molecules. We found that the PI3K/Akt/ERK/CREB(Snail)/MMP pathway was activated by CTHRC1, which modulated EMT change and invasion/metastasis in HCC. Intriguingly, CTHRC1 expression resulted in phosphorylations of Akt at the T308, S473, and S474 residues in HepG2 and HLK-3 cells. PI3K seemed to be upstream of Akt, but not PDK1 in HepG2 cells. This data was consistent with previous results that PI3 kinase/Akt promoted cell migration and invasion through the induction of MMP proteins and modulation of MMP expression [28, 31, 32]. CREB is located downstream of Akt, which induces phosphorylation of CREB Ser-133 and then stimulates the expression of the target genes via the activation of CREB [33]. Akt stimulates recruiting of CREB-binding protein (CBP) to CREB as well as phosphorylation of CREB. It is known that CREB induces metastasis of melanoma via MMP2 and adhesion molecules such as MCAM/MUC18 [34] and dominant-negative mutant CREB has been shown to suppress metastasis [35]. KG-501 binding to the transcription coactivator CBP blocks the interaction between CBP and phospho-CREB [33]. Our results revealed that KG-501 did not change AKT and CREB phosphorylation but decreased the expression of MMP2 and MMP9.

Another mechanism by which CTHRC1 promotes tumor invasion and metastasis may be related to EMT progression. It has been documented that Snail expression promoted migration and invasion in hepatocellular carcinoma [18]. In the present study, Snail protein expression was significantly increased in stable CTHRC1-HepG2 transfectants and in HepG2 cells transduced with Ad-CTHRC1, but not changed in Slug and Twist expression. Snail expression only modulated MMP9 expression in HepG2 cells. We also found increased expression of CTHRC1 after TGF-β1 and EGF treatment in HepG2 and SH-J1 cells and that growth factor inhibitors efficiently suppressed CTHRC1 expression and inhibited activation of their downstream molecules. Accordingly, it has been reported that upregulation of CTHRC1 by TGF-β1 was associated with metastasis in human gastric cancer [23].

In conclusion, CTHRC1 expression promoted cancer cell invasiveness and metastasis through activation of the PI3K/Akt/ERK/CREB (Snail) pathways, which induced EMT change and MMP expression. Therefore, CTHRC1 and its downstream molecules may be potential therapeutic targets in HCC progression, such as invasion and metastasis.

## MATERIALS AND METHODS

### Tissue samples

Pairs of HCC tissues and corresponding non-tumor liver tissues were acquired from patients at the Chonbuk National University Hospital. Written informed consent was obtained from each patient. Surgically removed tissues were examined for histological diagnosis and the remains were immediately snap-frozen in liquid nitrogen. Pathologists histologically confirmed HCC and non-tumor tissues. All protocols followed the guidelines of the Institutional Review Board (IRB).

### Western blotting

Proteins in the culture medium of cell lysates were displayed in 12% SDS-PAGE and were electrophoretically transferred to immobile transfer membranes (Millipore, Bedford, MA). Transferred blots were incubated with a blocking solution containing 5% dry milk in TBST [0.15% Tween 20, 200 mmol/L NaCl, and 25 mmol/L Tris-HCl (pH 7.6)]. Blots were washed and incubated for 1 h with secondary peroxidase-linked antibodies (Santa Cruz, CA). Immunoreactive bands were detected using ECL detection reagents (Amersham, Buckinghamshir, UK) according to the manufacturer's instructions.

For the non-reducing condition, cell lysates were resuspended in lysis buffer without DTT. Protein samples were then mixed with SDS sample buffer without reducing agents (2% SDS, 10% glycerol, 0.01% bromophenol blue and 62.5 mM Tris–Cl pH 6.8), and the samples were not boiled prior to loading on a 10% SDS–PAGE.

### Real-time PCR

Real-time PCR was completed using an ABI sequence detection system (Applied Biosystems, Foster City, CA). CTHRC1 primer sequence was used as previously described [24]. Experiments were performed in triplicate. The TaqMan 18S rRNA gene assay was used to normalize the relative abundance of mRNA.

### Immunohistochemistry and immunofluorescence

Immunohistochemistry staining was performed on formalin-fixed, paraffin-embedded tissue sections. After rehydration, a sample section was deparaffinized and pretreated by microwave epitope retrieval (750 W for 15 min). The primary rabbit antibody was used for the detection of CTHRC1 (DAKO, Glostrup, Denmark). Counter-staining was carried out using Meyer`s hematoxylin. Immunohistochemical staining of CTHRC1 was evaluated by the sum of the intensity scores and the area scores for each specimen. The intensity level of cytoplasmic and membranous staining was graded into four levels: no immunostaining (0), weak or trace (1), moderate (2), and strong (3). The proportion of positive staining cells was scored as follows: 0 (none), 1 (less than 1%), 2 (2% to 10%), 3 (11% to 33%), 4 (34% to 66%), and 5 (67% to 100%). If the final score was greater than 4 by totaling intensity scores and staining proportion, the immunoreactivity was considered positive. For negative controls, sections were treated with Tris-buffered saline instead of primary antibodies.

For immunofluorescence, HLK-3 and HepG2 cells were transfected with a GFP-tagged CTHRC1 expression vector or an empty vector control on glass coverslips, and then fixed with 4% paraformaldehyde in PBS with 0.2% Triton-X 100. Cells were then blocked for an hour with 1% bovine serum albumin (BSA) followed by incubation with rabbit polyclonal CTHRC1 antibody (1:100 dilution in PBS) overnight at 4°C. Cells were washed and incubated with TRITC-conjugated swine anti-rabbit antibody (DAKO). After a final wash, the cells were stained with 1 μg/ml Hoechst 33258 for 15 min, and the slides were mounted with 50% glycerol at 4°C.

### Phospho-proteome profiling

Cells were solubilized in NP-40 lysis buffer by rocking the lysates at 4°C for 30 minutes. Following microcentrifugation at 14,000 × g for 5 minutes, supernatants were transferred to a clean tube and then determine the protein concentrations using the Pierce Protein Assay Kit (Rockford, IL). Lysates (500 μg) were diluted and incubated with human phospho kinase arrays (R&D Systems, Minneapolis, MN).

### Gelatin zymography

Cells (1 × 10^6^) were seeded in 10-cm culture plates for 16 hours and cultured in serum-free medium for 72 hours. Conditioned medium was concentrated by centrifugation. Equal amounts of protein mixed with non-reducing Laemmli sample buffer were separated by SDS-PAGE containing 0.1% (w/v) gelatin. After incubation, MMP2 and MMP-9 activity was measured by Coomassie blue staining.

### Soft agar colony formation assay

Cells (5 × 10^4^) were plated in 60 mm plates in growth medium containing 0.7% agar (5 ml per well) on the top of growth medium containing 1.4% agar (3 ml per well). Growth medium (500 μl) with 10% FBS was added on top of the agar. The cell suspension was plated and cultured in a 37°C incubator for two weeks. After two weeks, viable colony formation was observed using an optical microscope.

### Luciferase report assay

Transcriptional activity assays were carried out using the Luciferase Assay System (Promega Corp, Medison, WI) according to the manufacturer`s instructions. HepG2 cells were simultaneously transfected with reporter plasmids pGL3B-MMP9, pGL3B-MMP2, pGL3B-MMP2-delSP1 [12] and expression plasmids pcDNA 3.1/myc HisA CTHRC1 or empty vector. Luciferase activity was assessed using a dual-luciferase reporter (DLR) Assay Kit (Promega) as instructed by the supplier. Luciferase activities were measured for normalization using an illuminometer (Lumat LB9507, Berthold, Bad Wildbad, Germany).

### Cell migration assay

Cells were grown to 90% confluence, rinsed with PBS and then starved for 36 hours in serum-free medium. A sterile pipette tip was used to make parallel wounds and migration across the wound line was measured after 48 hours. Three independent experiments were performed.

### *In vivo* metastasis assay

A tail vein injection assay was used to assess the effect of CTHRC1 on tumor metastasis. HepG2 cells stably expressing CTHRC1 or control cells that were infected with adeno-luciferase virus (Ad-CMV-Luc; Vector Biolabs, Philadelphia, PA) were injected into the tail veins (5 × 10^5^ cells in 200 μl PBS per mice). Mice were assessed for long-distance lung metastasis at 5 weeks (all 7 mice per group). The numbers of lung metastasis nodules were counted to analyze effects on spontaneous tumor metastasis.

### Bioluminescence imaging and analysis

CTHRC1-HepG2 cells infected with Ad-CMV-Luc or SH-J1 cells stably expressing luciferase (SH-J1-Luc) which were transduced with lentivirus encoding shRNA of CTHRC1 were used in an orthotopic nude mice lung metastasis tumor model. Briefly, Cells (5 × 10^5^ cells in 200 μl of culture medium) were injected into the tail veins of Balb/c mice (6 weeks old, n = 6). Tumor growth was monitored twice after 5 weeks by the Xenogen US/IVIS imaging system 100 (Caliper Lifescience, Hopkinton, MA). Mice were anesthetized with 3% isofuorance and intraperitoneally administrated with firefly D-luciferin, 150 mg/kg body weight, (Caliper Lifescience) for imaging.

### India ink lung staining

At 9 weeks after tail vein injection, mice were euthanatized and India ink (15%) was injected into their both lungs through the trachea. The lungs were fixed using Fekete's solution (5 mL glacial acetic acid, 10 mL formalin and 100 mL of 70% alcohol) at room temperature. After destaining with Fekete's solution, the lungs were photographed.

### Statistical analyses

Statistical analyses were carried out using GraphPad v.5.01. Correlations between CTHRC1 expression and clinicopathologic parameters was evaluated with the Mann-Whitney test or Kruskal-Wallis test. Differences were analyzed with dependent or independent *t*-tests. *P* values less than 0.05 were significant.

## SUPPLEMENTARY MATERIALS FIGURES AND TABLE



## Abbreviations

α-SMA: alpha-smooth muscle actin
CK8: cytokeratin 8
CK18: cytokeratin 18
CREB: cAMP response element-binding
CTHRC1: Collagen triple helix repeat containing 1
DesI/II: desmoplakin I/II
E-cad: E-cadherin
EGF: epidermal growth factor
EMT: epithelial-to-mesenchymal transition
FN: fibronectin
HCC: hepatocellular carcinoma
MMP: Matrix metalloproteinase
MTT: tetrazoliumsalt3-(4,5-dimethylthiazol-2-yl)-2,5-diphenylte trazolium bromide
N-cad: N-cadherin
TGF-β1: transforming growth factor-β1
VIM: vimentin

## References

[1] El-Serag HB, Rudolph KL (2007). Hepatocellular carcinoma: epidemiology and molecular carcinogenesis. Gastroenterology.

[2] Lau WY, Lai EC (2008). Hepatocellular carcinoma: current management and recent advances. Hepatobiliary Pancreat Dis Int.

[3] Pyagay P, Heroult M, Wang Q, Lehnert W, Belden J, Liaw L, Friesel RE, Lindner V (2005). Collagen triple helix repeat containing 1, a novel secreted protein in injured and diseased arteries, inhibits collagen expression and promotes cell migration. Circ Res.

[4] LeClair RJ, Durmus T, Wang Q, Pyagay P, Terzic A, Lindner V (2012). Cthrc1 is anovel inhibitor of transforming growth factor-beta signaling and neointimal lesion formation. Circ Res.

[5] Yamamoto S, Nishimura O, Misaki K, Nishita M, Minami Y, Yonemura S, Tarui H, Sasaki H (2008). Cthrc1 selectively activates the planar cell polarity pathway of Wnt signaling by stabilizing the Wnt-receptor complex. Dev Cell.

[6] Tang L, Dai DL, Su M, Martinka M, Li G, Zhou Y (2006). Aberrant expression of collagen triple helix repeat containing 1 in human solid cancers. Clin Cancer Res.

[7] Chen YL, Wang TH, Hsu HC, Yuan RH, Jeng YM (2013). Overexpression of CTHRC1 in Hepatocellular Carcinoma Promotes Tumor Invasion and Predicts Poor Prognosis. PloS One.

[8] Gialeli C, Theocharis AD, Karamanos NK (2011). Roles of matrix metalloproteinases in cancer progression and their pharmacological targeting. FEBS J.

[9] Roy R, Yang J, Moses MA (2009). Matrix metalloproteinases as novel biomarkers and potential therapeutic targets in human cancer. J Clin Oncol.

[10] Deryugina EI, Quigley JP (2006). Matrix metalloproteinases and tumor metastasis. Cancer Metastasis Rev.

[11] Blackburn JS, Liu I, Coon CI, Brinckerhoff CE (2009). A matrix metalloproteinase-1/protease activated receptor-1 signaling axis promotes melanoma invasion and metastasis. Oncogene.

[12] Xia L, Huang W, Tian D, Zhu H, Zhang Y, Hu H, Fan D, Nie Y, Wu K (2012). Upregulated FoxM1 expression induced by hepatitis B virus X protein promotes tumor metastasis and indicates poor prognosis in hepatitis B virus-related hepatocellular carcinoma. J Hepatol.

[13] Park JK, Park SH, So K, Bae IH, Yoo YD, Um HD (2010). ICAM-3 enhances the migratory and invasive potential of human non-small cell lung cancer cells by inducing MMP-2 and MMP-9 via Akt and CREB. Int J Oncol.

[14] van Zijl F, Zulehner G, Petz M, Schneller D, Kornauth C, Hau M, Machat G, Grubinger M, Huber H, Mikulits W (2009). Epithelial-mesenchymal transition in hepatocellular carcinoma. Future Oncol.

[15] Jou J, Diehl AM (2010). Epithelial-mesenchymal transitions and hepatocarcinogenesis. J Clin Invest.

[16] Giannelli G (2009). The epithelial-mesenchymal transition: fact or fiction in cancer. Hepatology.

[17] Teoh NC (2010). Pre-”EMT”ing key processes in liver carcinogenesis. growing evidence for how malignant hepatocytes invade and conquer. Hepatology.

[18] Yang MH, Chen CL, Chau GY, Chiou SH, Su CW, Chou TY, Peng WL, Wu JC (2009). Comprehensive analysis of the independent effect of twist and snail in promoting metastasis of hepatocellular carcinoma. Hepatology.

[19] Giannelli G, Bergamini C, Fransvea E, Sgarra C, Antonaci S (2005). Laminin-5 with transforming growth factor-beta1 induces epithelial to mesenchymal transition in hepatocellular carcinoma. Gastroenterology.

[20] Ding W, You H, Dang H, LeBlanc F, Galicia V, Lu SC, Stiles B, Rountree CB (2010). Epithelial-to-mesenchymal transition of murine liver tumor cells promotes invasion. Hepatology.

[21] Best JL, Amezcua CA, Mayr B, Flechner L, Murawsky CM, Emerson B, Zor T, Gardner KH, Montminy M (2004). Identification of small-molecule antagonists that inhibit an activator: coactivator interaction. Proc Natl Acad Sci U S A.

[22] Park EH, Kim S, Jo JY, Kim SJ, Hwang Y, Kim JM, Song SY, Lee DK, Koh SS (2013). Collagen triple helix repeat containing-1 promotes pancreatic cancer progression by regulating migration and adhesion of tumor cells. Carcinogenesis.

[23] Wang P, Wang YC, Chen XY, Shen ZY, Cao H, Zhang YJ, Yu J, Zhu JD, Lu YY, Fang JY (2012). CTHRC1 is upregulated by promoter demethylation and transforming growth factor-beta1 and may be associated with metastasis in human gastric cancer. Cancer Sci.

[24] Tan F, Liu F, Liu H, Hu Y, Liu D, Li G (2013). CTHRC1 is associated with peritoneal carcinomatosis in colorectal cancer: a new predictor for prognosis. Med Oncol.

[25] Ma MZ, Zhuang C, Yang XM, Zhang ZZ, Ma H, Zhang WM, You H, Qin W, Gu J, Yang S, Cao H, Zhang ZG (2014). CTHRC1 acts as a prognostic factor and promotes invasiveness of gastrointestinal stromal tumors by activating Wnt/PCP-Rho signaling. Neoplasia.

[26] Kim HC, Kim YS, Oh HW, Kim K, Oh SS, Kim JT, Kim BY, Lee SJ, Choe YK, Kim DH, Kim SH, Chae SW, Kim KD (2014). Collagen triple helix repeat containing 1 (CTHRC1) acts via ERK-dependent induction of MMP9 to promote invasion of colorectal cancer cells. Oncotarget.

[27] Cheng JC, Chou CH, Kuo ML, Hsieh CY (2006). Radiation enhanced hepatocellular carcinoma cell invasion with MMP-9 expression through PI3-Kinase/Akt/NF-kB signal transduction pathway. Oncogene.

[28] Bae IH, Park MJ, Yoon SH, Kang SW, Lee SS, Choi KM, Um HD (2006). Bcl-w promotes gastric cancer cell invasion by inducing matrix metalloproteinase-2 expression via phosphoinositide 3-kinase, Akt, and Sp1. Cancer Res.

[29] Yoeli-Lerner M, Toker A (2006). Akt/PKB signaling in cancer: a function in cell motility and invasion. Cell Cycle.

[30] Chung TW, Lee YC, Kim CH (2004). Hepatitis B viral HBx induces matrix metalloproteinase-9 gene expression through activation of ERK and PI-3K/Akt pathways: involvement of invasive potential. FASEB J.

[31] Weng CJ, Chau CF, Hsieh YS, Yang SF, Yen GC (2008). Lucidenic acid inhibits PMA-induced invasion of human hepatomacells through inactivating MAPK/ERK signal transduction pathway and reducing binding activities of NF-kappaB and AP-1. Carcinogenesis.

[32] Yoeli-Lerner M, Toker A (2006). Akt/PKB signaling in cancer: a function in cell motility and invasion. Cell Cycle.

[33] Du K, Montminy M (1998). CREB is a regulatory target for the protein kinase Akt/PKB. J Biol Chem.

[34] Jean D, Bar-Eli M (2000). Regulation of tumor growth and metastasis of human melanoma by the CREB transcription factor family. Mol Cell Biochem.

[35] Aucoin R, Reiland J, Roy M, Marchetti D (2004). Dominant negative CREB inhibits heparanase functionality and melanoma cell invasion. J Cell Biochem.

