# Supplementary material for: CTHRC1 activates pro-tumorigenic signaling pathways in hepatocellular carcinoma
Source: Oncotarget. 2017 Oct 27;8(62):105238–50. doi: 10.18632/oncotarget.22164 (PMC5739634; doi:10.18632/oncotarget.22164)
Supplement: Supplementary file 1 [file oncotarget-08-105238-s001.pdf]

CTHRC1 activates pro-tumorigenic signaling pathways in hepatocellular carcinoma

SUPPLYMENTARY METARIALS

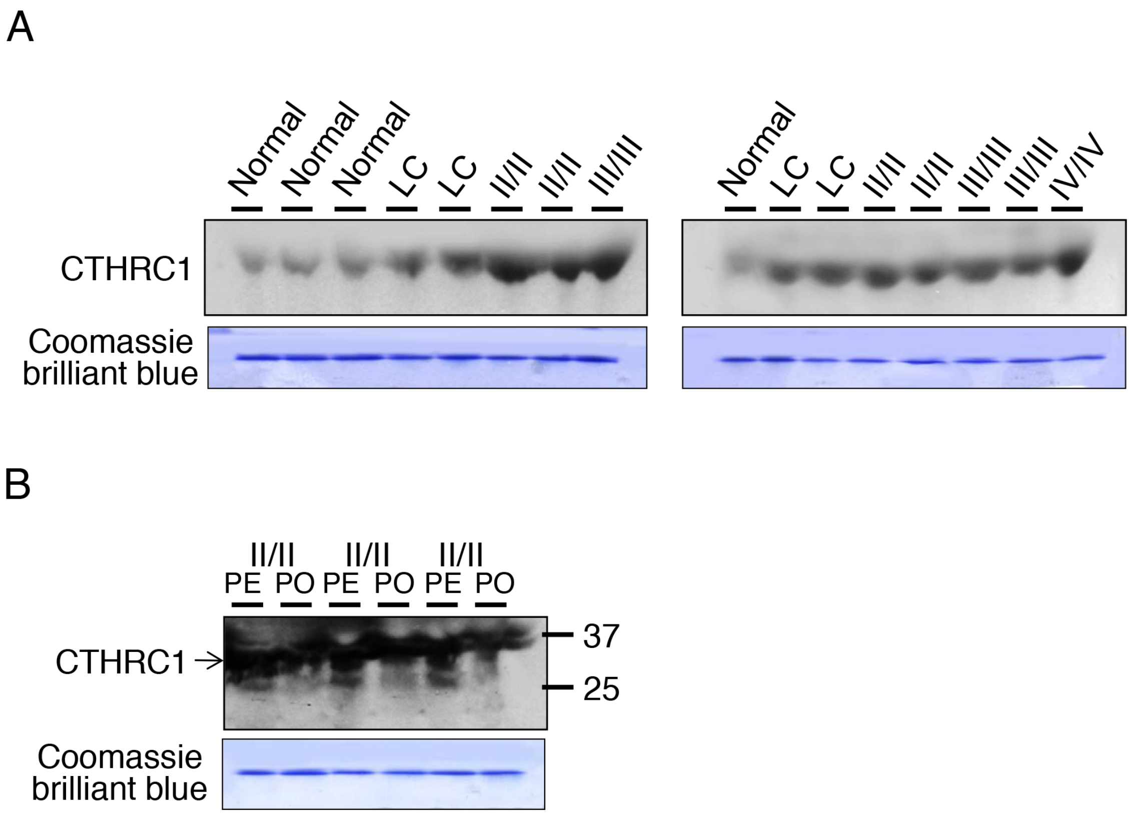

Supplementary Figure 1: Western blot analysis of secreted CTHRC1 expression in the serum of HCC patients. (A) High expression of secreted CTHRC1 in the serum of HCC patients compared with normal control serum. **(B)** Secreted CTHRC1 in the serum of HCC patients was decreased after surgery (PE: presurgery, PO: postsurgery).

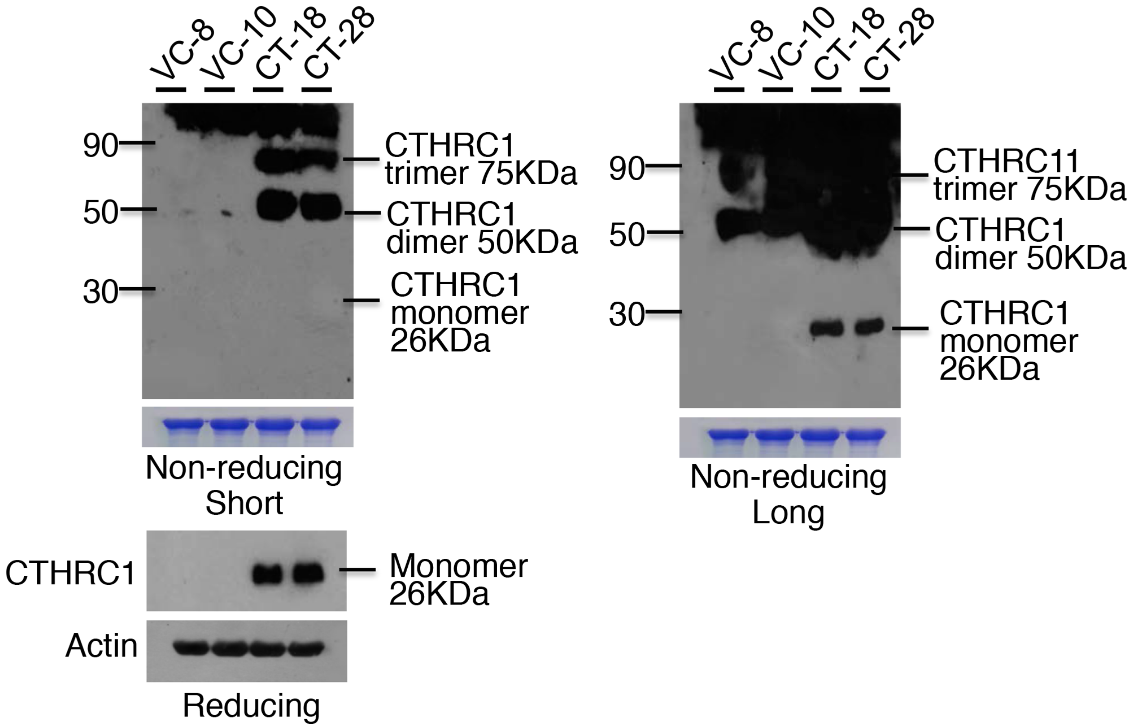

Supplementary Figure 2: Western blot analysis of secreted CTHRC1 protein in the conditioned medium of HepG2 cells. Samples were resolved by 12% SDS–PAGE under non-reducing condition, after which the blot was probed with anti-CTHRC1 antibodies. The three forms of CTHRC1 were 26, 50, and 75 kDa in size, corresponding to CTHRC1 monomers, homodimers, and homotrimers, of CTHRC1, respectively. Short, short exposure; Long, long exposure.

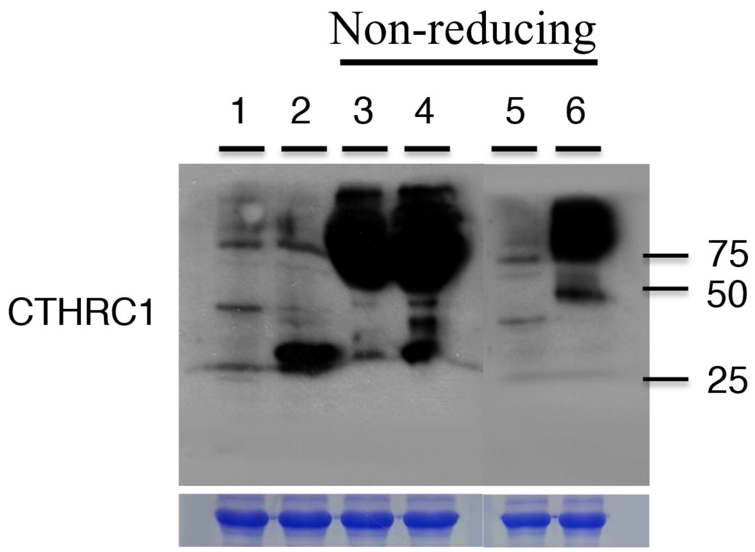

Supplementary Figure 3: Western blot analysis of CTHRC1 protein in the cell lysate and conditioned medium of HepG2 cells infected with adenovirus expressing CTHRC1. Only the 26 kDa form of CTHRC1 was detected in the cell lysate, whereas three forms of CTHRC1 were detected in the conditioned medium under non-reducing condition. The three forms of CTHRC1 were 26, 50, and 75 kDa, corresponding to CTHRC1 monomer, homodimer, and heterodimeric, respectively (lane 1, Ad-LacZ cell lysate; lane 2, Ad-CTHRC1 cell lysate; lane 3, Ad-LacZ cell supernatant ; lane 4, Ad-CTHRC1 cell supernatant; lane 5, Ad-LacZ cell lysate; lane 6, Ad-CTHRC1 cell lysate).

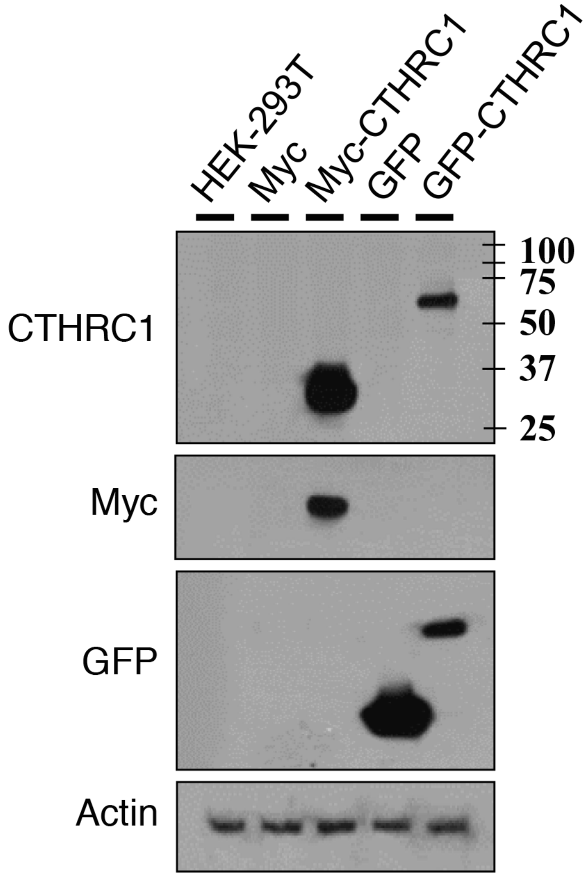

Supplementary Figure 4: Specific immunoreactivity for CTHRC1. Specific immunoreactivity of anti-CTHRC1 against CTHRC1 determined by Western blot analysis. Protein lysates were prepared from HEK293T cells transfected with either GFP or c-Myc vector alone or GFP- or c-Myc-tagged CTHRC1. The membranes were incubated with anti-GFP, anti-Myc, or anti-CTHRC1 antibodies. The numbers on the right correspond to molecular weight markers.

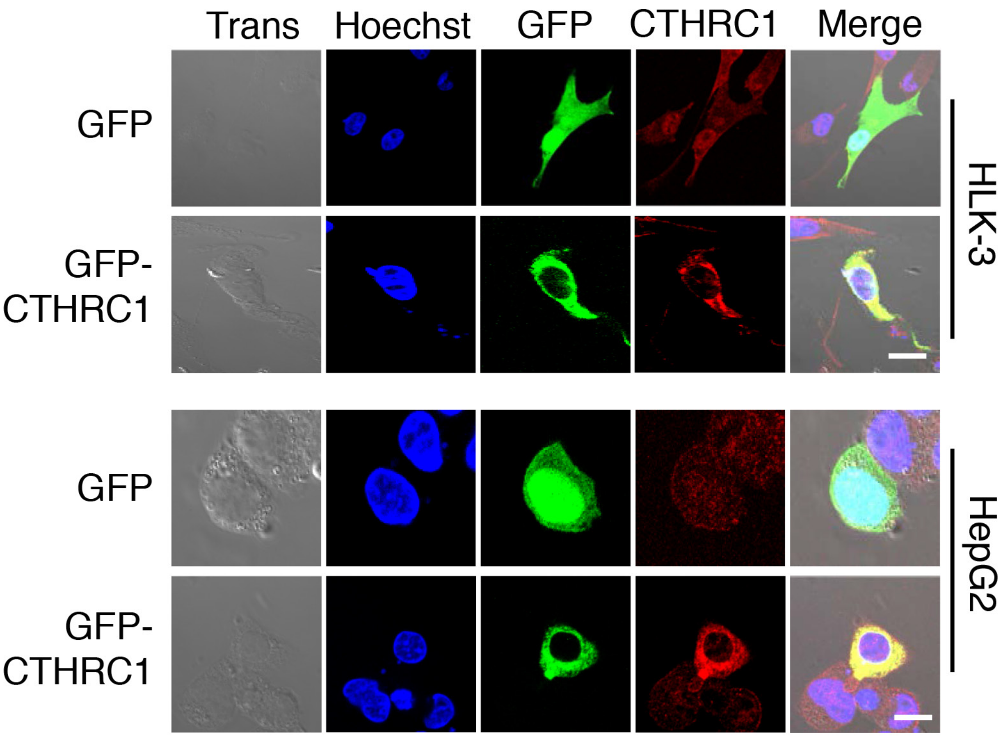

Supplementary Figure 5: Cellular localization of CTHRC1. HLK-3 and HepG2 cells were transfected with the GFP-tagged CTHRC1 expression vector (GFP-CTHRC1) or empty vector control (GFP), and antibody immunoreactivity (red) was assayed by immunofluorescence. Trans, transmission; Bar, 20 µm.

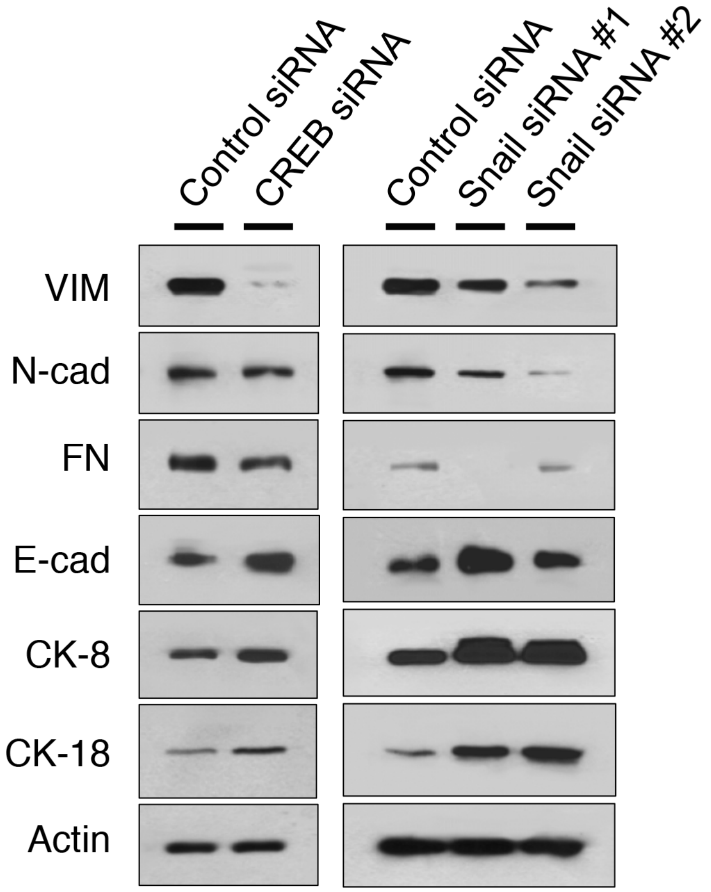

Supplementary Figure 6: Changes of EMT maker expressions by CREB or snail. Western blot analyses showed an acquisition of epithelial and a loss of mesenchymal marker proteins after transfection with siRNA of CREB or Snail, compared with control siRNA in HepG2 cells. VIM, vimentin; N-cad, N-cadherin; FN, fibronectin; E-cad, E-cadherin; CK, cytokeratin

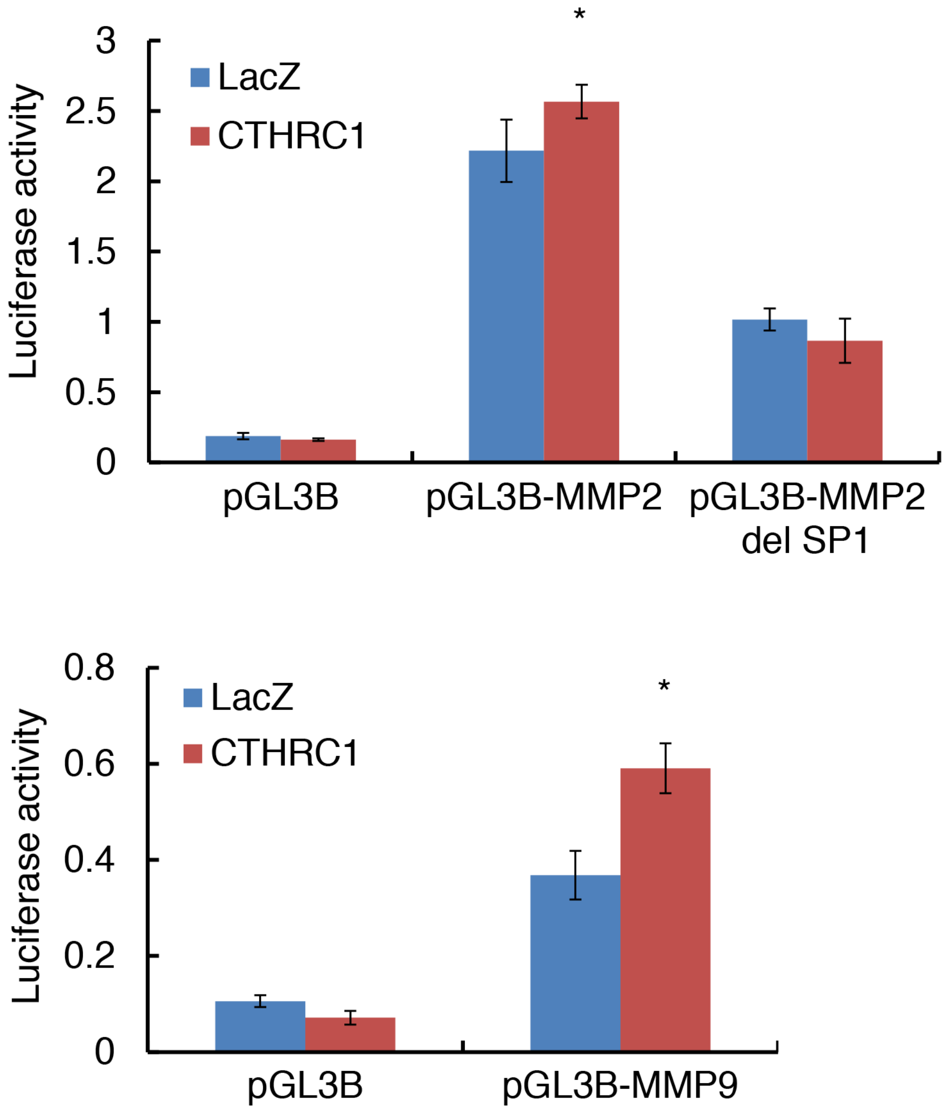

Supplementary Figure 7: Transcriptional promotion of MMP by CTHRC1. Transcriptional regulation of MMP2 was assessed in HepG2 cells using a luciferase reporter (pGL3B-MMP2-luc, pGL3B-MMP2 del SP1-luc) and a control (pGL3B) (upper panels). Luciferase reporter (pGL3B-MMP9-luc) and pGL3B were used for the assay of MMP9 transcriptional regulation.(lower panels). Each value represents the mean ± SEM from three independent experiments in duplicate; $^{*}P < 0.05$.

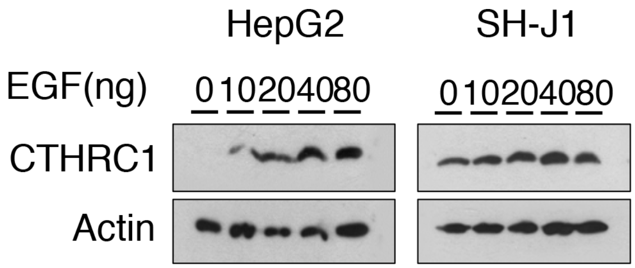

Supplementary Figure 8: EGF-induced CTHRC1 expression in HepG2 and SH-J1 cells. Cells were treated with EGF at the indicated concentrations for 4 days.

Supplementary Table 1: Correlation of *CTHRC1* expression with various clinicopathological features of HCC specimens by real-time RT-PCR analysis

		Total	Median	Mean	Lower 95% CI of mean	Upper 95% CI of mean	*P*-Value
Age (median)	≤58	65	4.773	14.01	8.597	19.42	.7877
	>58	63	4.363	15.46	9.253	21.66	
Sex	Male	110	4.568	13.86	9.643	18.08	.7164
	t	18	4.821	19.98	6.059	33.9	
HBV	Positive	73	3.407	13.58	8.492	18.67	.0806
	Negative	55	6.454	16.24	9.478	23	
Liver cirrhosis	yes	39	2.094	11.8	4.143	19.45	$.0394^{*}$
	no	89	5.917	15.78	11.06	20.5	
Tumor size(cm)	<3	52	1.943	9.676	4.236	15.12	$.0034^{**}$
	3~5	46	1.346	16.65	9.389	23.91	
	>5	30	7.433	20.52	10.87	30.17	
ES Grade	I	8	0.8285	1.117	0.4238	1.811	$<.0001^{***}$
	II	51	3.407	10.37	5.594	15.14	
	III	63	6.604	18.69	11.68	25.7	
	IV	6	24.32	28.23	11.31	45.03	
Recurrence	yes	53	7.181	19.22	11.75	26.7	.1025
	no	75	4.116	11.63	7.343	15.92	
Tumor number	1	117	4.363	15.14	10.81	19.46	.3743
	>1	11	4.773	10.33	1.69	22.36	
Fibrous capsule formation	yes	96	3.583	13.1	6.527	14.05	.0653
	no	32	7.108	19.58	10.15	29.01	
Capsular infiltration	yes	67	4.116	15.23	9.346	21.1	.8918
	no	61	5.917	14.17	8.456	19.88	
Microvessel invasion	yes	66	4.8	19.91	12.97	26.85	$.0457^{*}$
	no	62	3.592	9.197	5.521	12.87	
Intrahepatic metastasis	yes	27	10.52	30.18	17.63	42.73	$.0051^{**}$
	no	101	3.451	10.59	6.957	14.22	
Stage (AJCC)	I/II	100	3.114	9.418	5.997	12.84	$<.0001^{***}$
	III/IV	28	24.27	32.95	21.33	44.57	
AFP	<200	90	3.583	10.9	6.859	14.94	.0545
	≥200	36	7.829	19.89	10.91	28.87	
	nd	2					

HBV, hepatitis B virus; AFP, serum alpha-fetoprotein.
Differences between subgroups were tested by Mann-Whitney or Kruskal-Wallis tests.
$^{*}p < 0.05$; $^{**}p < 0.001$; $^{***}p < 0.0001$. nd, not determined.
